# The Future of Hospitals: A Disclaimer

**Published:** 1910-08-20

**Authors:** G. A. Heron


*To the Editor of* The Hospital.

Yours faithfully,

George W. F. Robbins, Secretary.

### THE FUTURE OF HOSPITALS : A DISCLAIMER.

*To the Editor of* The Hospital.

Sir,—In your issue of August 6, on p. 565, I am reported as having said that a certain statement, publicly made and in my hearing, by Mr. Sydney Holland . . . "was one of the most disgraceful a man had ever made in public." I do not think I used those words. If I did use them they were certainly an exaggeration of what I intended to say. My reference to Mr. Holland's speech was an *impromptu*, suggested to me by something said by a former speaker in the discussion.

The words I have quoted from your report are not only an exaggeration, they are also discourteous. For those reasons I should feel obliged, whether or not the words have been ascribed in error to me, if you would, by publishnig this letter, give me an opportunity of expressing my regret that the words have appeared in a report of a speech for which, in some measure, I am responsible.

The remainder of your report of what I said at that meeting of the Section of Medical Sociology is in substance correct, and shows, I hope, clearly that in my opinion Mr. Sydney Holland's statement concerning medical men who undertake unpaid work in public hospitals was insulting to the honorary medical officers of hospitals, and unworthy of a man occupying Mr. Holland's high position in the hospital work of London.

It was only to-day that I first saw The Hospital of August 6, hence delay on my part in sending you this letter.

I am, sir, yours faithfully,

August 10, 1910. G. A. Heron.

